# Expert-Based Narrative Review on Compression UltraSonography (CUS) for Diagnosis and Follow-Up of Deep Venous Thrombosis (DVT)

**DOI:** 10.3390/diagnostics15010082

**Published:** 2025-01-02

**Authors:** Mario D’Oria, Laura Girardi, Ahmed Amgad, Mohab Sherif, Gabriele Piffaretti, Barbara Ruaro, Cristiano Calvagna, Philip Dueppers, Sandro Lepidi, Marco Paolo Donadini

**Affiliations:** 1 Division of Vascular and Endovascular Surgery, Department of Clinical Surgical and Health Sciences, University of Trieste, 34127 Trieste, Italy; 2 Research Center on Thromboembolic Diseases and Antithrombotic Treatment, Department of Medicine and Surgery, University of Insubria, 21100 Varese, Italy; 3 Faculty of Medicine, Helwan University, Cairo 11792, Egypt; 4 Vascular Surgery, Department of Medicine and Surgery, School of Medicine, University of Insubria, 21100 Varese, Italy; 5 Department of Pulmonology, University Hospital of Cattinara, 34149 Trieste, Italy; 6 Department of Vascular Surgery, Kantonsspital St. Gallen, 9000 St. Gallen, Switzerland

**Keywords:** compression ultrasound, deep vein thrombosis, diagnostic techniques, duplex ultrasound, venous thromboembolism, post-thrombotic syndrome, prognosis, residual vein obstruction

## Abstract

Deep venous thrombosis (DVT) is a pathological condition that develops when a thrombus forms within the deep venous system. Typically, it involves the lower limbs and, less frequently, the upper extremities or other unusual districts such as cerebral or splanchnic veins. While leg DVT itself is rarely fatal and occasionally can lead to limb-threatening implications, its most fearsome complication, namely pulmonary embolism, is potentially fatal and significantly contributes to increased healthcare costs and impaired quality of life in affected patients and caregivers. Thanks to its high accuracy, ease of use, and safety profile, duplex ultrasound (DUS), particularly compression ultrasound (CUS), has emerged as the first-line imaging modality for DVT diagnosis. The evaluation of suspected DVT needs a multifaceted approach, and in this context, CUS rapidly became a key diagnostic tool owing to its many unique advantages. Its central role in the diagnostic algorithm of suspected DVT is clearly established in the latest clinical practice guidelines from the European Society for Vascular Surgery and the American Society of Haematology. Indeed, DUS effectively visualizes blood flow and identifies abnormalities like clot formation with high sensitivity (typically exceeding 90% for proximal DVT) and specificity (often approaching 100% for proximal DVT). Additionally, CUS is non-invasive, readily available at the bedside, and avoids radiation exposure, resulting in an ideal method for various clinical settings. CUS has been shown to have a substantial role not only in the diagnosis of an acute DVT but also in the follow-up of its management. Moreover, this method can provide a prognostic assessment, mostly in terms of risk stratification for recurrent thrombosis and/or for potential complications, such as post-thrombotic syndrome. In summary, given its established benefits, CUS is a technique that many physicians should be familiar with, especially those working in emergency departments, intensive care units, or general wards. When needed, healthcare operators with more advanced US skills (such as radiologists, angiologists, or vascular surgeons) may be called upon to provide a second look in case of uncertainty and/or need for additional information.

## 1. Introduction

Deep venous thrombosis (DVT) is a pathological condition that develops when a thrombus forms within the deep venous system. Typically, it involves the lower limbs and, less frequently, the upper extremities (mainly in the context of thoracic outlet syndrome or in patients with long-standing central venous catheterization) [1] or other unusual districts such as cerebral or splanchnic veins. While leg DVT itself is rarely fatal and occasionally can lead to limb-threatening implications, its most fearsome complication, namely pulmonary embolism (PE), is potentially fatal and significantly contributes to increased healthcare costs and impaired quality of life in affected patients and caregivers. According to available evidence, around 33% of DVT cases may eventually progress to PE if left untreated [2,3]. Early DVT diagnosis and prompt intervention are therefore crucial to preventing this serious outcome [2]. Furthermore, another frequent significant long-term complication of DVT is post-thrombotic syndrome (PTS). In fact, PTS may affect up to 20–50% of DVT patients and is characterized by several signs and symptoms, including chronic pain, edema, and skin discoloration, until the development of venous ulcers in the affected limb [4,5].

Thanks to its great accuracy, ease of use, reproducibility, tolerability, and optimal safety profile, duplex ultrasound (DUS), particularly compression ultrasound (CUS), has emerged as the first-line imaging modality for DVT diagnosis [6]. Indeed, DUS effectively visualizes blood flow and identifies abnormalities like clot formation with high sensitivity (typically exceeding 90% for proximal DVT) and specificity (often approaching 100% for proximal DVT) [7]. Additionally, CUS is non-invasive, readily available at the bedside, and avoids radiation exposure, resulting in an ideal method for various clinical settings [6].

This narrative review aims to explore the contemporary evidence on the use of CUS for the diagnosis and follow-up of DVT. By means of a multidisciplinary expert-based assessment of the available evidence, this work will encompass the topic in a brief yet comprehensive manner, therefore providing clinicians with an up-to-date reference, as well as identifying gaps in the literature that may deserve further research efforts.

## 2. Overview of CUS Technique for Diagnosis of DVT: Pearls and Pitfalls

### 2.1. Principles of CUS

**Figure 1:**
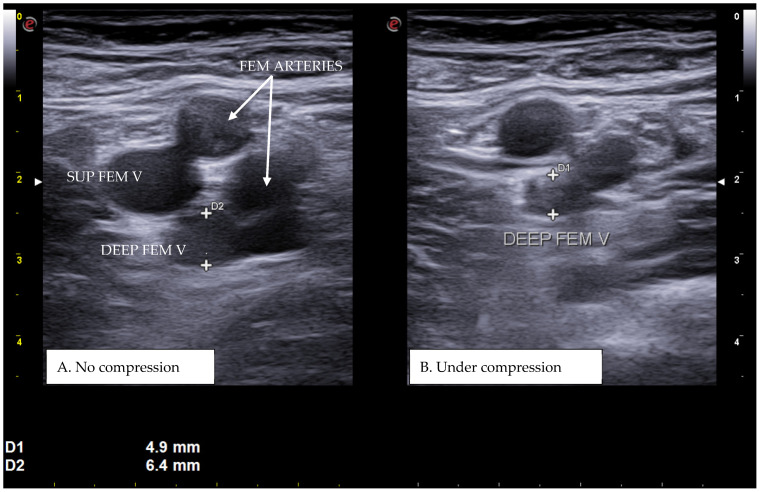
A real example of deep femoral vein thrombosis at the level of the upper left thigh (groin region). D1 shows the residual diameter of the non-compressible vein (i.e., the maximum diameter of the relative venous thrombus). D2 represents the venous diameter without compression.

**Figure 2:**
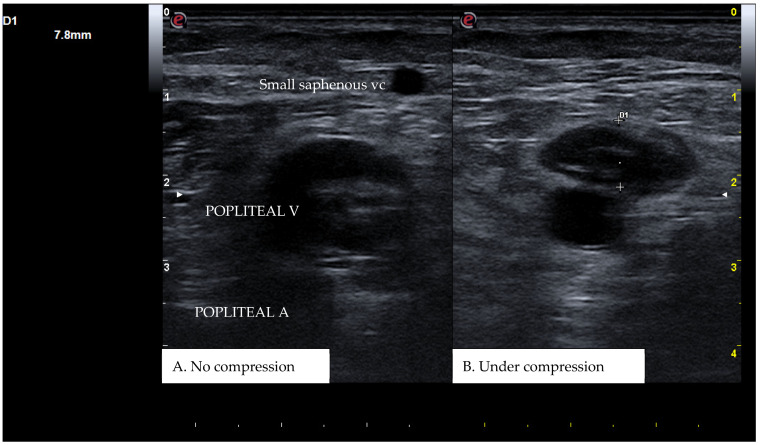
A real example of popliteal vein thrombosis at the level of the left popliteal cavity. D1 shows the residual diameter of the non-compressible vein (i.e., the maximum diameter of the relative venous thrombus).

**Figure 3:**
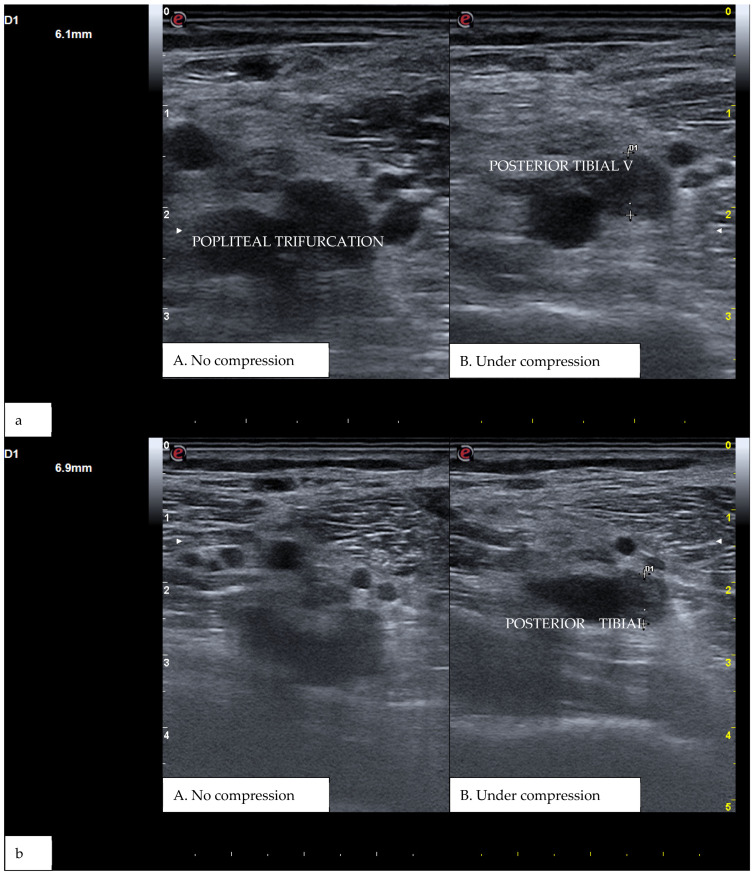
(**a**) A real example of DVT at the level of the right popliteal trifurcation with posterior tibial vein involvement. (**b**) Represents the same patient at a more distal section. D1 shows the residual diameter of the non-compressible vein (i.e., the maximum diameter of the relative venous thrombus).

**Figure 4:**
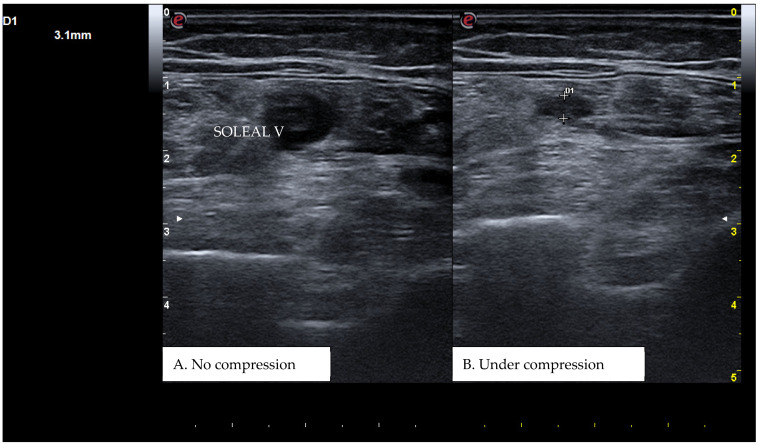
A real example of soleal vein thrombosis at the level of the right distal popliteal region. D1 shows the residual diameter of the non-compressible vein (i.e., the maximum diameter of the relative venous thrombus).

CUS is a diagnostic technique primarily designed to find DVT by checking the veins’ compressibility with the principle that healthy veins are normally compressible and would collapse under the pressure of the ultrasonographic probe. In the case of the presence of a venous thrombus, the vessel would not be able to collapse under such pressure [8,9]. Linear high-frequency transducers are generally used for CUS as they best visualize leg veins [10]. The ultrasound machine settings must be adjusted carefully in order to limit possible operator-related bias. Important elements to be considered in this setting include gain, focus, and time-gain compensation. Gain adjustment regulates image brightness, focus optimization sharpens the image to the desired depth, and time-gain compensation enhances the visibility of subtle echoes, ensuring uniform image quality [10,11]. The transducer partially compresses the vein, which creates an echogenic response. In normal veins, this technique should fully compress the lumen [9]. However, with thrombosis present, the vein does not collapse enough, which causes a non-continuous echogenic line [12]. While the femoral veins are usually well identified in the groin and thigh areas, to facilitate better visibility of the popliteal veins, the patient may lie in a supine position with the leg slightly abducted to relax the muscles or, alternatively, in a prone position [9]. Real-time B-mode and Doppler imaging are both valuable tools in this process. B-mode imaging can find incomplete vein collapse during compression [13,14]. Doppler imaging can show the thrombus or find a complete lack of flow in the lumen [9]. As such, CUS for DVT diagnosis involves evaluating direct signs like the presence of a non-compressible echogenic line suggesting a thrombus, as well as indirect signs like changes in vein compressibility and thrombus echogenicity [6,7]. In addition, evaluation of flow patterns and flow response during augmentation maneuvers may refine the diagnostic examination, although their implementation requires more advanced training and skills than simple CUS [7]. Clinicians should assess a spectrum of veins, including the external iliac, common femoral, superficial and deep femoral, popliteal, tibial, peroneal, gastrocnemial, and soleal veins, as well as the junctions with the great and small saphenous veins, representing the superficial systems [15]. Challenges exist in visualizing the iliac veins, especially in obese patients, yet phasic flow in the femoral veins can be indicative of DVT status, particularly for excluding iliac vein thromboses [16]. This comprehensive approach is standard in differentiating healthy veins, which should collapse and enlarge with augmentation maneuvers, from those with thrombosis, which show impaired response to pressure and flow changes [12,17]. Figure 1, Figure 2, Figure 3 and Figure 4 show real examples of DVT affecting different sites of the lower limb deep venous system, diagnosed through a CUS modality.

### 2.2. Advantages of CUS

In acute DVT cases, an experienced practitioner can distinguish between the features of old and new thrombi with relative ease and accuracy [18]. Early after the onset of DVT, a non-compressible vein is filled by mostly non-echogenic material, which is, therefore, interpreted as an acute thrombus. Because of the vascular occlusion, there may be either no or very few flow signals received in the color Doppler mode [18,19,20]. On the other hand, post-thrombotic sequelae, such as residual vein obstructions, also called and known as “chronic thrombus”, will usually be visualized as a well-collateralized vessel that is at least partially compressible and sometimes with a typical “dual-rail” sign of the vessel wall. There may be low-level echoes due to the fibrotic component of the old thrombus [18,19,20]. In color Doppler mode, there may or may not be flow signals, depending on how much the affected vein has recanalized [12].

As already noted, CUS is non-invasive and lacks ionizing radiation, which may be seen as the main advantage of this diagnostic modality when compared with other techniques like venography, the former gold standard, or computed tomography scanning, making this examination well suited also for repeated assessments over time [9,21]. Indeed, ultrasound is now firmly established by evidence-based clinical practice guidelines as the first-line imaging study for diagnostic assessment of venous thrombosis for both inpatients and outpatients. Furthermore, CUS has emerged as a cost-effective diagnostic tool for suspected DVT due to its economic advantages, especially for repeated screenings and follow-ups [8].

Another distinct advantage of the ultrasound-based technique is the possibility of utilizing a single probe to switch between B-mode, C-mode, and PW-mode [10,11,22]. This capability allows for a holistic examination, beginning with the visualization of venous and surrounding anatomy in B-mode to identify any suspicious features, including, for instance, the potential identification of external compression by masses or enlarged lymph nodes that may signal a concomitant pathology [10,11,22]. Transitioning to C-mode enables the assessment of blood flow presence and characteristics, such as respiratory phasicity and response to activation maneuvers [10,11,22]. Additionally, shifting into PW-mode offers a detailed analysis of flow dynamics [10,11,22]. The option to alter presets from venous to arterial facilitates the evaluation of arterial perfusion, which is particularly vital for the diagnosis of severe conditions like “phlegmasia cerulea dolens”, where arterial blood flow is critically impaired [23].

### 2.3. Limitations and Pitfalls of CUS

A key factor that affects the accuracy of CUS for the diagnosis of DVT, along with other pathologies, is represented by the operators’ skill level. Current guidelines for CUS recommend that the exam should be performed by a skilled provider; however, the exact definition of the level of training required for performing state-of-the-art CUS is currently lacking [24]. Patients’ habitus may also be riddled with additional challenges. Obese patients, for example, represent a growing population, and this condition might pose a pitfall in the diagnosis of DVT. In fact, in addition to the technical difficulty of performing a correct compression maneuver in this setting, the increased amount of subcutaneous tissue limits the ultrasound beam penetration and affects the image quality [25]. Therefore, both the sensitivity and specificity of CUS are reported to decrease by a range of 10% to 60% and 3% to 25%, respectively, in the obese patient population [26]. Besides obesity, the literature also points to the limitations in visualizing and compressing the iliac veins in the pelvic region using standard CUS. Variations in venous anatomy and location of the left common iliac vein may hamper visualization and identification of a thrombus in this district [16]. Pregnancy, especially during the third trimester owing to the enlarged uterus and presence of ascites, constitutes a typical condition that may further impair ultrasound accuracy in this context.

These findings demonstrate that although CUS can be regarded as the recommended first-line imaging assessment for DVT, great emphasis should be on the operator’s capability and awareness of anatomical or physiological pitfalls. As examples of the latter condition, there are some anatomical areas that typically make it difficult to achieve complete vein compression by the probe, limiting, therefore, CUS accuracy, for instance, above the inguinal ligament in the pelvis region where iliac veins are located, or in the lower and mid-thigh and the lower calf where the compression is limited by the absence of rigid structures under the veins, or by proximity to the bone, respectively. In these cases, the use of color Doppler modality can be of further help. Alternatively, especially when DVT is suspected to be a consequence of underlying conditions such as malignant neoplasms, or when an evaluation of the cava vein is required, additional imaging techniques like computed tomography angiography (CTA) and magnetic resonance imaging (MRI) may provide a detailed and comprehensive view of the venous system and its surrounding structures, enhancing the diagnostic process by offering insights into both the thrombotic condition itself and any associated pathologies [27,28].

The challenge of diagnosing recurrent DVT further complicates the clinical picture, presenting a scenario where the distinction between new, acute-on-chronic thrombotic events and the remnants of previous thromboses, such as webs or scars, becomes crucial yet challenging [6,29]. In these instances, the reliance on CUS may be limited, and the role of advanced diagnostic tools, including intravascular ultrasound (IVUS) or venography, may become important [9,30]. These modalities, along with careful clinical evaluation and consideration of D-dimer levels, though nonspecific, are critical in differentiating acute thrombotic processes from chronic post-thrombotic changes [31]. Such a complex diagnostic approach is essential for establishing the presence of active thrombosis and determining the most appropriate management plan [32].

## 3. Indications for CUS in the Diagnostic Algorithm of DVT

### 3.1. Clinical Presentation of and Risk Factors for Lower Extremity DVT

Although the presentation of DVT can vary among different patients, certain clinical features are frequently observed. The most reported sign is leg swelling [33,34], usually on one side, which involves the calf and/or, less frequently, the thigh. The swelling may come with pain, often described as throbbing or cramping [31]. Moreover, the affected area might feel warm and might show erythema or discoloration [34]. Some patients may also present with visible dilated superficial veins in the extremity [33]. However, it is crucial to acknowledge the heterogeneity in DVT presentation. Not everyone will have all the typical signs and symptoms, with a non-negligible proportion of patients reporting no symptoms [33,34]. This shows the importance of having a high suspicion of DVT in the workup of alternative diagnoses [31].

Researchers have identified several established risk factors that may predispose individuals to DVT. We can categorize these factors as transient or permanent conditions, some of which can be inherited through genetic transmission [5]. Transient risk factors represent a wide range of clinical scenarios, including major surgery, prolonged immobilization, and major trauma. Additionally, chronic conditions such as cancers, chronic inflammation, heart failure, and autoimmune diseases may significantly elevate the risk of developing a DVT [5]. Autoimmune diseases such as systemic lupus erythematosus, rheumatoid arthritis, and antiphospholipid syndrome represent some of the most common acquired conditions associated with an increased clotting tendency [30,35]. Other acquired risk factors include pregnancy, hormonal therapies, and obesity [5].

From both a pathogenetic and a prognostic perspective, it is especially relevant to classify DVT as either unprovoked or provoked and to distinguish the latter based on the presence of either transient or permanent risk factors. This initial evaluation is crucial since it will help determine the long-term risk for recurrence and the subsequent need for prolonged anticoagulation [4,5,32].

Inherited conditions, also known as congenital thrombophilias, represent a particular group of heterogeneous genetically-based disorders that significantly contribute to DVT risk by promoting an excessive tendency to clot [5]. The most well-known are the Factor V Leiden mutation and the Prothrombin G20210A mutation. Factor V Leiden, the most common inherited form of thrombophilia in people of European descent, results from a mutation that causes Factor V to be resistant to deactivation by activated protein C, leading to an increased risk of clot formation [5], whereas the Prothrombin G20210A mutation increases the levels of prothrombin [5]. Other, less common, inherited thrombophilias include a significant reduction in natural anticoagulants such as Protein C, Protein S, and Antithrombin deficiencies. These proteins normally help regulate blood clotting, and their deficit can lead to an increased risk of forming abnormal blood clots [5].

Identifying these conditions is crucial for effectively managing treatment options and prevention strategies, especially among individuals with a strong family history of thrombosis [32,36]. Understanding both acquired and congenital risk factors is essential for developing a comprehensive risk assessment and management strategy for patients susceptible to DVT [32,36]. As recommended by the European Society for Vascular Surgery (ESVS) guidelines, screening for thrombophilia or cancer should be individualized based on the presentation of each patient by taking into consideration several factors, such as age, sex, and family history of thrombosis, among others.

### 3.2. Incorporation of CUS in the Diagnostic Workup of Suspected DVT

The evaluation of suspected DVT needs a multifaceted approach, and in this context, CUS has emerged as a key diagnostic tool owing to its many unique advantages. Its central role in the diagnostic algorithm of suspected DVT is clearly established in the latest clinical practice guidelines from the European Society for Vascular Surgery and the American Society of Haematology [24,37]. In detail, the guidelines recommend a multimodal approach that incorporates a clinical assessment of pre-test probability, laboratory examinations, and duplex ultrasound [37]. Using prediction models such as the Wells score and other pre-test probability assessments to raise suspicion of DVT is, in fact, generally recommended [31,34,37]. More precisely, the Wells score can estimate the chance of a patient having DVT. The likelihood can be graded as either low (score < 2), moderate (score 2–6), or high (score > 7) [33,34]. CUS is then recommended as a first-line imaging procedure for patients with a moderate to high pre-test probability, with an indication to repeat imaging after 5–7 days if negative at first assessment [17,31]. Conversely, when the pre-test probability is low, measurement of D-dimer is recommended thanks to its high negative predictive value [33,37]. If the D-dimer is positive, CUS is recommended. In the presence of a negative D-dimer, DVT can instead be safely ruled out with no need to undergo further imaging, and no repeat examination is usually warranted [33,37]. Only when a duplex ultrasound remains inconclusive or not feasible are second-line imaging techniques recommended (including CTA, MRI, and venography) [37,38].

### 3.3. Evaluation of DVT in Post-Operative and Hospitalized Patients

In bedridden, post-operative, and hospitalized patients, acute DVT may have an atypical presentation or even ensue with no overt symptoms. Furthermore, D-dimer may be elevated owing to several surgical or medical diseases, thereby making its clinical utility poor. In this scenario, CUS is safe and effective for diagnosing DVT and can be performed at the bedside as needed to rule out the disease [31,37].

## 4. Diagnostic Accuracy of CUS (Two-Point vs. Three-Point CUS; Proximal vs. Distal DVT)

Over the last three decades, the use of CUS has been significantly increasing, becoming the reference standard in most diagnostic algorithms and also being included in the point-of-care US (POCUS) examinations of patients in emergency departments. Indeed, CUS has shown an average sensitivity for proximal DVT of 97% and an average specificity of 98%. Moreover, standard CUS has a shorter learning curve as compared to other US techniques and may be easily performed at the bedside [39]. However, some differences may exist in terms of diagnostic accuracy when considering two-point or three-point proximal vein CUS or whole-leg US (also named in the literature as “complete” lower limb ultrasound or “comprehensive” duplex ultrasonography). Indeed, physicians may either perform a limited examination of the common femoral vein and the popliteal vein (two-point CUS) or examine the entire proximal venous system, from the common femoral vein to the popliteal vein and its trifurcation (three-point CUS). Both techniques are considered acceptable in the context of a diagnostic workup of DVT based on previously determined pre-test clinical probability and, when indicated, D-dimer. Nonetheless, in the context of suspected DVT, this simplified US technique that scans proximal veins only may underdiagnose some distal thromboses. Therefore, such an approach may require the repetition of a CUS after 5–7 days, which could be inconvenient for patients and physicians to assess for propagation of undetected distal DVT. Alternative strategies have been proposed over the years to obviate imaging repetition, including the performance of whole-leg US, which encompasses all venous segments from the iliac to the calf veins. A systematic review and meta-analysis combining the results of seven studies was performed to evaluate the safety of using a single whole-leg CUS to exclude DVT after an initially normal result [40]. The VTE event rate at 90 days was 0.57% (95% CI 0.25–0.89%), which is considered safe in the context of DVT diagnostic strategies [40]. Indeed, a 2% false negative rate is considered the post-test probability threshold for an acceptable diagnostic pathway [24].

Since whole-leg CUS is more time-consuming and requires a longer learning curve and adjunctive skills, another approach has been tested in a diagnostic management trial by using limited CUS in case of either high pre-test probability or positive D-dimer, without repeating CUS after 7 days, and whole-leg CUS in case of both high pre-test probability and elevated D-dimer. Overall, the 3-month VTE incidence in patients left untreated after a negative diagnostic workup was very low, reported to be 0.87% (95% CI 0.44–1.70) [41]. Therefore, this strategy may offer significant advantages in the management of patients with suspected DVT.

## 5. The Role of CUS in the Follow-Up of DVT

CUS has been shown to have a substantial role not only in the diagnosis of an acute DVT but also in the follow-up of its management. Moreover, this method can provide a prognostic assessment, mostly in terms of risk stratification for recurrent VTE and/or for potential complications, such as PTS. Above all, the repetition of CUS at the end of a proper cycle of anticoagulant treatments for acute DVT (baseline imaging), or at any time during such therapies, can give important information in terms of the grade of vessel recanalization, the identification of potential progression of the thrombus, diagnostic confirmation of recurrent events, and detection of potential complications related to treatments (e.g., subcutaneous or muscle hematomas), in case of clinical suspicion. All these details are crucial for clinicians when they are faced with the decision of establishing the duration of anticoagulation, which is the result of an accurate balance of individual thrombotic and bleeding risks.

Over the last decades, several studies have tried to identify the potential role of ultrasound detection of residual vein thrombosis (RVT), also called residual vein obstruction (RVO), mostly evaluated as a potential independent risk factor for recurrent VTE. Although there is no standardized metric for RVO, its definition and classification can vary depending on different US parameters, of which the most widely used is the evidence of a residual venous non-compressibility of at least 3 mm [42]. In a systematic review and meta-analysis, RVO was shown to be significantly associated with recurrent thrombotic events in patients with any (unprovoked or provoked) DVT (OR 1.5, 95% CI 1.1–2.0) [43]. These results align with another systematic review on the topic [44]. Conversely, a recent management study conducted on 825 consecutive patients diagnosed with acute, symptomatic, proximal DVT of the lower extremity showed that RVO was independently associated with PTS and arterial events but not with venous recurrence or cancer [45]. In a patient-level meta-analysis, the association between RVO and recurrent events was shown to be present, and it was stronger if RVO was detected at an earlier time (at the 3-month mark) after the index thrombosis [42]. Similar conclusions are shown by the most recent systematic review on the topic, where, following an unprovoked DVT, ultrasound determination of RVO was mildly predictive of VTE recurrence, but only when assessed soon after the index thrombotic event (i.e., 3 months) [46]. Therefore, current evidence on the efficacy and utility of the identification of RVO as an element on which to base a decision on a tailored therapy duration is still scarce and controversial. Although a consensus is generally found on the inclusion of RVO among useful factors to be evaluated for individual patient risk stratification, clinical guidelines suggest against routine use of US to detect RVT after a first unprovoked event [36]. Decision-making on the duration of anticoagulation should be based on additional elements, such as the presence of permanent risk factors (e.g., active cancer) and the pathogenesis of the index event (e.g., unprovoked) [36].

More uniformity is found in the association of RVO with the development of PTS. Indeed, PTS is a common complication of DVT that can have different grades of severity and consequent impact on patients’ quality of life. Leg pain, edema, and skin discoloration are the most frequent elements leading patients to frequent clinical reassessments. Unfortunately, there are no specific treatment strategies available for PTS as its management relies on tools and techniques generally used for chronic venous disease, including elastic compression of the lower limb, physical activity, and endovascular recanalization of occluded venous segments. Therefore, the best option remains to provide constant implementation of prevention and early identification of high-risk patients. A systematic review and meta-analysis evaluated potential US-detectable predictors of PTS [47]. The authors identified two US parameters that seemed to be predictive of PTS, namely venous reflux at the popliteal vein (PVR) and residual thrombosis measured at least 6 weeks after DVT [47]. Furthermore, recent evidence reported that the use of compressive stockings for the prevention of PTS was associated with better outcomes among patients with no evidence of RVT and/or PVR compared to those showing these US findings at the 6-month mark after a proximal DVT [48]. Based on the known potential diagnostic bias derived from operators’ skills, a prospective cohort study was performed with the aim of evaluating the interobserver reliability of the measurement of residual thrombosis in patients with a first unprovoked DVT of the leg at the six-month mark [49]. The results of this analysis were very promising in the identification, measurement, and percentage assessment of residual obstruction, confirming the extreme utility of this imaging technique for this setting [49].

Aside from prognostic importance, a baseline US for evaluation of venous recanalization is also crucial for the correct diagnosis of potential subsequent recurrent events. According to the second consensus updated document elaborated in 2022 by the working group on the aorta and peripheral vascular diseases of the European Society of Cardiology, venous US assessment, prior to anticoagulation discontinuation, is useful in determining baseline residual vein thrombosis not to drive anticoagulant treatment duration, but to differentiate between old and new thrombosis in case of new symptoms [50]. Indeed, the definition of recurrent DVT detected with CUS includes (a) a new non-compressible vein in the contralateral leg, (b) a new non-compressible vein of the same leg as the index event, that was previously unaffected, (c) a significant proximal extension of the known thrombus, or (d) a new non-compressible site of a vein that was previously affected, as long as there had been an interim US showing resolution of the index event [42], or an increased non-compressibility of > 4 mm of the index event [24]. Therefore, the repetition of CUS, especially at the time of completion and discontinuation of treatments, is a key point for discrimination between the diagnosis of recurrent events and residual symptoms more likely to be related to PTS.

## 6. Conclusions: Summary of Findings, Current Controversies, and Future Directions

CUS has become the diagnostic test of choice for detecting DVT, resulting in its widespread use across almost all clinical contexts, including outpatient clinics, emergency departments, and other hospital divisions. Its diagnostic accuracy is optimal in clinical scenarios of suspected DVT if combined with algorithms comprising pre-test probability assessments and D-dimer measurements. Appropriate operator skills are needed, especially to discriminate those situations in which the examination may prove suboptimal and second-line imaging modalities may be needed. Nonetheless, differences exist between the limited CUS (2-point or 3-point) and the extended CUS (whole-leg) in terms of a learning curve, yield, and execution time. Therefore, a number of strategies have been proposed and tested in clinical trials, especially to minimize the risk of underdiagnosing distal thrombosis and, at the same time, to eliminate or reduce the need for imaging repetition after 5–7 days. Anyhow, a case-to-case evaluation is recommended to obtain the best results both from a clinical and cost-effectiveness standpoint. Furthermore, CUS may be very important in patients with suspected PE who may have absolute or relative contraindications to undergo computed tomography pulmonary angiography (CTPA), for example, pregnant women, patients with severe renal failure, or those with a history of severe reaction to iodine contrast medium. In these clinical scenarios, the possibility of performing an easy, non-invasive test such as CUS can lead to the diagnosis of VTE and the start of treatment even without the need for CTPA [24,51]. This strategy has also been positively tested in pregnant women in recent years. Moreover, CUS can be incorporated in a multi-POCUS model, including lung US and focused cardiac US, to improve PE diagnostic strategies and reduce the use of inappropriate requests for CTPA [42,52,53]. Finally, CUS has proved to be accurate in detecting residual thrombosis after primary treatment of DVT (3–6 months), thus providing important data to inform clinical decisions [54]. Indeed, even if RVO should not be considered as a stand-alone parameter to decide about the optimal duration of anticoagulation, it may be included in a case-by-case evaluation, among other important decision parameters (e.g., type and causes of index event, sex, age, previous VTE events, and bleeding risk assessment) [55]. Additionally, performing CUS to detect RVO at the time of anticoagulation withdrawal is crucial to correctly evaluate the patient in case of possible subsequent suspicion of recurrent ipsilateral DVT. In addition to those roles of RVO, its assessment is also important for the diagnosis of post-thrombotic syndrome and the choice of the best treatment [56].

In summary, given its established benefits, CUS is a technique that many physicians should be familiar with, especially those working in emergency departments, intensive care units, or general wards. When needed, healthcare operators with more advanced US skills (such as radiologists, angiologists, or vascular surgeons) may be called upon to provide a second look in case of uncertainty and/or need for additional information. Some questions remain that may pave the way for future research.

## Abbreviations

CTA	Computed Tomography Angiography
CUS	Compression Ultrasound
DUS	Duplex UltraSound
DVT	Deep Vein Thrombosis
MRI	Magnetic Resonance Imaging
PE	Pulmonary Embolism
POCUS	Point-Of-Care Ultrasound
PTS	Post-Thrombotic Syndrome
RVO	Residual Vein Obstruction
RVT	Residual Vein Thrombosis
VTE	Venous Thromboembolism
